# Is the transperineal ultrasonography approach effective for the diagnosis of rectocele?

**DOI:** 10.1093/gastro/goab019

**Published:** 2021-05-29

**Authors:** Yi-Bo Yao, Hao-Qiang Yin, Hai-Jun Wang, Hong-Tao Liang, Bo Wang, Chen Wang

**Affiliations:** 1 Department of Proctology, Longhua Hospital affiliated to Shanghai University of TCM, Shanghai, P. R. China; 2 Department of Ultrasonic, Longhua Hospital affiliated to Shanghai University of TCM, Shanghai, P. R. China; 3 Shanghai Hao Zhuo Data Service Co. Ltd, Shanghai, P. R. China

**Keywords:** transperineal ultrasonography, defecation proctography, rectocele, sitting position, squatting position, consistency

## Abstract

**Background:**

Transperineal ultrasonography has been used as a diagnostic imaging modality for rectocele for many years. However, the consistency of ultrasonography and defecography in evaluating the severity of rectocele was not satisfactory. This study aimed to evaluate the agreement in the measurement of rectocele parameters between the two methods in different positions and provide clinical implications for the diagnosis of rectocele.

**Methods:**

In this pilot study, participants were recruited in an outpatient clinic of a tertiary hospital between December 2017 and December 2019. All participants separately underwent defecation proctography at sitting and squatting positions, and undertook transperineal ultrasonography at left lateral, sitting, and squatting positions. The consistency of ultrasonography and defecography was evaluated.

**Results:**

Thirty female volunteers with rectocele were included in this study. The degree of anorectal angle was significantly larger at rest and during contraction, maximal Valsalva, and evacuation; the depth of the rectocele was significantly deeper during maximal Valsalva and evacuation; and the length of the perineum descending was significantly longer during contraction and maximal Valsalva in using squatting position compared to the sitting position when performing the defecation proctography. The degree of anorectal angle, the depth of rectocele, the area of levator hiatus, and the volume of the rectocele were significantly different in using squatting, sitting, and left lateral positions when performing the transperineal ultrasonography. Bland-Altman semi-quantitative plots showed good consistency in the measurement of the anorectal angle and the depth of the rectocele between proctography and ultrasonography in both sitting and squatting positions.

**Conclusions:**

The findings of our study may be considered as the preliminary evidence to support the use of transperineal ultrasonography with sitting and squatting positions as the imaging test of choice for evaluating patients with rectocele.

## Introduction

Rectoceles are commonly found in conjunction with obstructed defecation and pelvic-floor dysfunction. It presents as an out pocketing of the anterior rectal and posterior vaginal wall into the lumen of the vagina, mostly occurring during defecation [1]. Rectocele is a common condition in females. These patients usually do not become symptomatic until the fourth or fifth decade of life [2]. Clinical diagnosis and evaluation of a woman with rectocele are based on her medical history, physical examination, and imaging modality [3, 4].

To date, the diagnostic gold standard for rectocele is defecation proctography, which is used to identify anatomical disorders in combination with the need for an extended defecogram, including contrast in the bladder and vagina as well as occasional peritoneography [5]. However, this approach is relatively costly with radiation exposure. In recent years, image diagnostic techniques like dynamic magnetic resonance imaging (MRI) and transperineal ultrasonography have been increasingly used to diagnose and evaluate defecatory disorders in clinical practice [6, 7]. The advantages of these imaging modalities are the absence of ionizing radiation, comfort, and non-invasiveness. Transperineal ultrasonography can also be used for the diagnosis of other pelvic-floor disorders linked to rectocele [8]. However, the consistency of these methods in the identification of rectocele diagnosis compared to defecation proctography was not satisfactory [9]. In addition, patients are asked to take a sitting position for the defecography test and take a supine position and/or left lateral position for the dynamic MRI and ultrasonography [10, 11].

The dynamic MRI method is costlier than the transperineal ultrasonography and defecation proctography techniques, and most previous studies have only examined the abnormal defecate position to scale the severity of the rectocele [12–14]. Therefore, we conducted this pilot study aiming to compare defecation proctography and transperineal ultrasound in different defecate positions regarding their consistency in the measurement of rectocele parameters.

## Materials and methods

### Study design and study subjects

This prospective observational study was carried out at the Longhua Hospital affiliated to Shanghai University of Traditional Chinese Medicine between December 2017 and December 2019. This study was approved by the local research ethics committee of Longhua Hospital (IRB No. 2017LCSY018). All procedures performed in studies involving human participants were in accordance with the ethical standards of the institutional review board at Longhua Hospital affiliated to Shanghai University of TCM and with the 1964 Helsinki Declaration and its later amendments or comparable ethical standards. Informed consent was obtained from all individual participants included in the study.

Participants meeting the clinical diagnosis of rectocele by digital rectal examination [15] were included in this study. The exclusion criteria included hemorrhoids, anal fissure, and rectal hemorrhage that predict cancer. For all participants, their demographic and clinical characteristics were collected including age, body mass index, reproductive history, favorite defecation position, and daily stool quality, obstructed defecation syndrome (ODS) scores [16], and constipation scoring system (CSS) [17]. These participants were provided with a Bristol stool chart to describe their daily stool quality [18].

### Defecation proctography protocol

**Figure 1.**
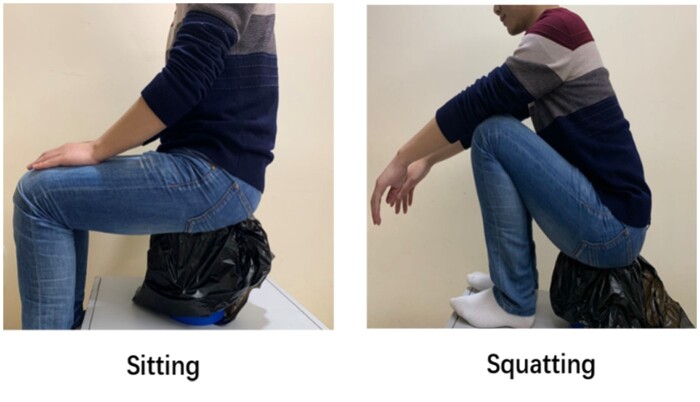
Different positions for defecation proctography (simulation)

Defecation proctography was performed by an experienced radiologist. The rectum was filled with 120 ml diluted barium sulfate. The volunteer was examined using a sitting position on a radiolucent commode on the first day then the same procedure was performed using a squatting position on the second day (SIEMENS IconosR200 Digital Gastrointestinal System) (Figure 1). Images were recorded in the sagittal plane at rest and during contraction, maximal Valsalva, and evacuation of contrast.

### Transperineal ultrasonography protocol

**Figure 2.**
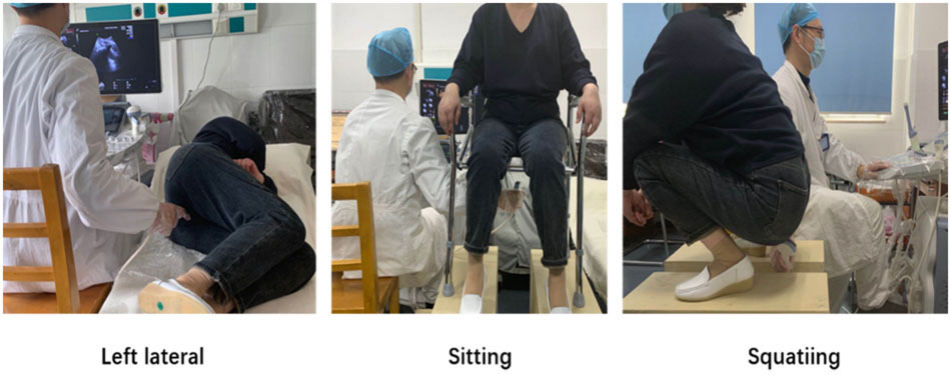
Different positions for transperineal ultrasonography (simulation)

Transperineal ultrasonography was performed by an experienced sonographer in the left lateral, sitting, and squatting positions (Figure 2) with a 1-hour interval with an empty bladder using a 1.5- to 6-MHz C1-6-D convex array probe and 5.0- to 9-MHz RIC-9-D real-time 4D probe (Voluson10 expert, GE Healthcare, Milwaukee, WI). The rectum was filled with a 100-ml ultrasonographic coupling gel mix with a 0.5-ml suspension of SF6 microbubbles at each position. The assessment was carried out in the midsagittal plane. Volumes were obtained at rest and during contraction, maximal Valsalva, and evacuation of contrast. Rectocele volume was calculated in contrast-enhanced ultrasound 3D mode, the probe scanned, and the volume was calculated automatically during maximal Valsalva.

### Outcome measurement

Data of the anorectal angle and depth of the rectocele of participants were obtained using both the defecation proctography and transperineal ultrasonography techniques. In addition, the perineum descending was measured when using the defecation proctography method, while the area of the levator hiatus and volume of the rectocele were determined when using the transperineal ultrasonography method. It is worth noting that all measurements of these two image diagnostic methods were administered by two different operators.

### Statistical analysis

The median and the interquartile range (IQR) were used to describe the measurements of anorectal angle, depth of rectocele, perineum descending, area of the levator hiatus, and the volume of the rectocele. The Wilcoxon signed-rank test and repeated measures ANOVA were used to compare the difference in measurements of anorectal angle, depth of rectocele, perineum descending, area of the levator hiatus, and volume of the rectocele between the defecography and ultrasonography groups, and across different position groups, as appropriate. Agreement test was used to detect the consistence between defecation proctography and transperineal ultrasonography in sitting and squatting positions, respectively. A Bland-Altman plot was used to show the appearance of differences between the different techniques. Paired *t*-test was used to compare the time performed for the two evaluations. The data were analysed using STATA 14.2 and the significant level was set at 0.05.

## Results

### Sample characteristics

A total of 30 Chinese female patients were recruited. The average age of the volunteers was 51.56 ± 14.48 years and the average body mass index was 22.73 ± 2.53 kg/m^2^. All participants were multipara with one or two eutocie. Nine had a previous hysterectomy. No patients reported vault prolapse. The median value of the Bristol stool quality among all participants was 5 (range, 2–6). The average ODS and CSS scores were 12.07 ± 5.88 and 11.67 ± 5.03, respectively. Four patients stated that their favorite defecation position was the squatting position.

### Defecation proctography

**Figure 3.**
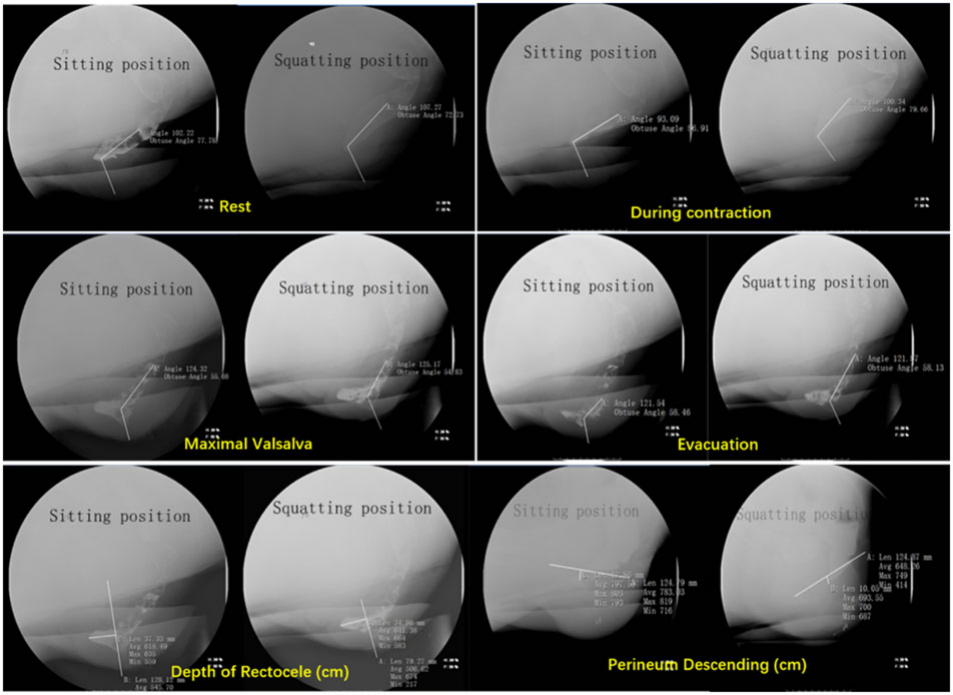
Anorectal angle (at rest and during contraction, maximal Valsalva, and evacuation), depth of rectocele (at maximal Valsalva), and perineum descending (at maximal Valsalva) by defecation proctography in two positions

The anorectal angle, depth of rectocele, and perineum descending images via the defecation proctography method are shown in Figure 3. Compared to the sitting position, participants in the squatting position showed a larger degree of anorectal angle at rest (*P *=* *0.023) and during contraction (*P *<* *0.001), maximal Valsalva (*P *<* *0.001), and evacuation (*P *<* *0.001); a deeper depth of rectocele at maximal Valsalva (*P *<* *0.001) and evacuation (*P *=* *0.004); and a longer length of perineum descending during contraction (*P *=* *0.002) and maximal Valsalva (*P *=* *0.022) (Table 1).

**Table 1.** Results of defecation proctography in two positions

Defecation photography		Sitting position (*n* = 30)	Squatting position (*n* = 30)	*P*-value
Anorectal angle, degree, median (IQR)	Rest	105 (100, 110)	107 (104, 112)	0.023
	During contraction	92 (89, 94)	97 (90, 102)	<0.001
	Maximal Valsalva	118 (112, 124)	125 (119, 134)	<0.001
	Evacuation	116 (109, 120)	119 (111, 126)	<0.001
Depth of rectocele, cm, median (IQR)	Rest	0 (0, 1)	0 (0, 1)	0.839
	During contraction	0 (0, 0)	0 (0, 0)	0.052
	Maximal Valsalva	2 (2, 3)	3 (3, 4)	<0.001
	Evacuation	2 (1, 3)	3 (2, 3)	0.004
Perineum descending, cm, median (IQR)	Rest	2 (1, 2)	2 (2, 2)	0.069
	During contraction	1 (1, 1)	1 (1, 1)	0.002
	Maximal Valsalva	4 (3, 4)	4 (3, 5)	0.022
	Evacuation	3 (3, 3)	3 (3, 4)	0.059

### Transperineal ultrasonography

**Figure 4.**
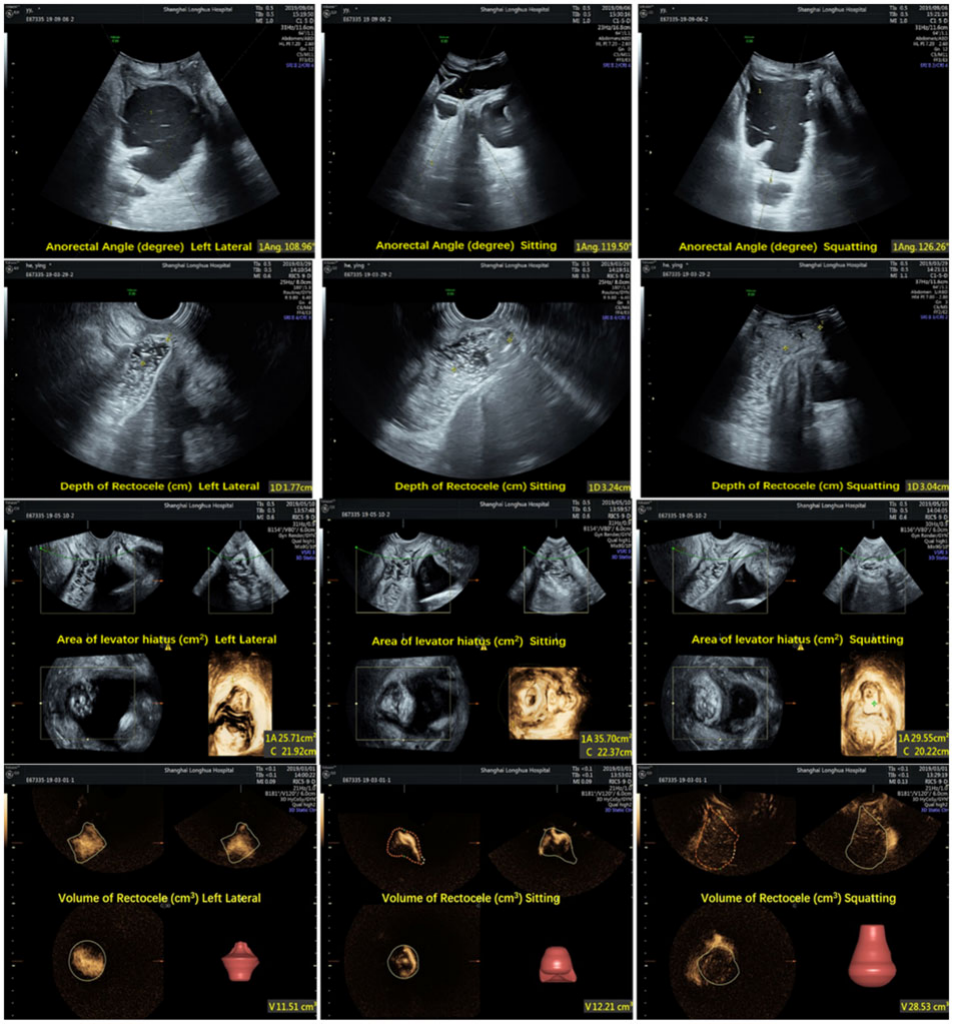
Anorectal angle, depth of rectocele, area of levator hiatus, and volume of rectocele (at maximal Valsalva) by transperineal ultrasonography in three positions

The anorectal angle, depth of rectocele, area of levator hiatus, and volume of rectocele images via the transperineal ultrasonography method are shown in Figure 4. There were statistically significant differences among the left lateral, sitting, and squatting positions in anorectal angles at rest (*P *<* *0.001); during contraction (*P *=* *0.002), maximal Valsalva (*P *<* *0.001), and evacuation (*P *<* *0.001); and in depth of rectocele during contraction (*P *=* *0.027), maximal Valsalva (*P *<* *0.001), and evacuation (*P *<* *0.001). Significant differences in the area of levator hiatus (*P *<* *0.001) and volume of rectocele (*P *=* *0.002) at maximal Valsalva had also been found among these three positions (**Table 2**).

**Table 2.** Results of transperineal ultrasonography in three positions

Transperineal ultrasonography		Left lateral position (*N* = 30)	Sitting position (*N* = 30)	Squatting position (*N* = 30)	*P*-value
Anorectal angle, degree, median (IQR)	Rest	98 (95, 101)	103 (100, 112)	105 (100, 111)	<0.001
	During contraction	88 (86, 91)	92 (88, 96)	91 (89, 95)	0.002
	Maximal Valsalva	117 (108, 121)	123 (116, 127)	124 (119, 131)	<0.001
	Evacuation	109 (104, 115)	118 (109, 121)	119 (111, 122)	<0.001
Depth of rectocele, cm, median (IQR)	Rest ^a^	0 (0, 0.9)	0 (0, 1.8)	0 (0, 1.6)	0.071
	During contraction ^a^	0 (0, 0)	0 (0, 0.3)	0 (0, 0.5)	0.027
	Maximal Valsalva	2 (2, 3)	2 (2, 3)	3 (2, 4)	<0.001
	Evacuation	0 (0, 0)	0 (0, 0)	0 (0, 1)	<0.001
Area of levator hiatus, cm^2^, median (IQR)^b^	Rest	21 (18, 26)	25 (20, 34)	23 (19, 30)	0.023
	Maximal Valsalva	32 (29, 35)	39 (32, 40)	37 (31, 41)	<0.001
Volume of rectocele, cm^3^, median (IQR)^b^	Maximal Valsalva	8 (7, 12)	10 (9, 12)	12 (9, 15)	0.002

a Given that all the interquartile ranges (IQRs) of the depth of the rectocele at rest and during contraction (0, 0), median (range) were used to describe these measurements.

b Results cannot be measured in some situations (e.g. during contraction).

### Comparison between the defecography and ultrasonography results

**Figure 5.**
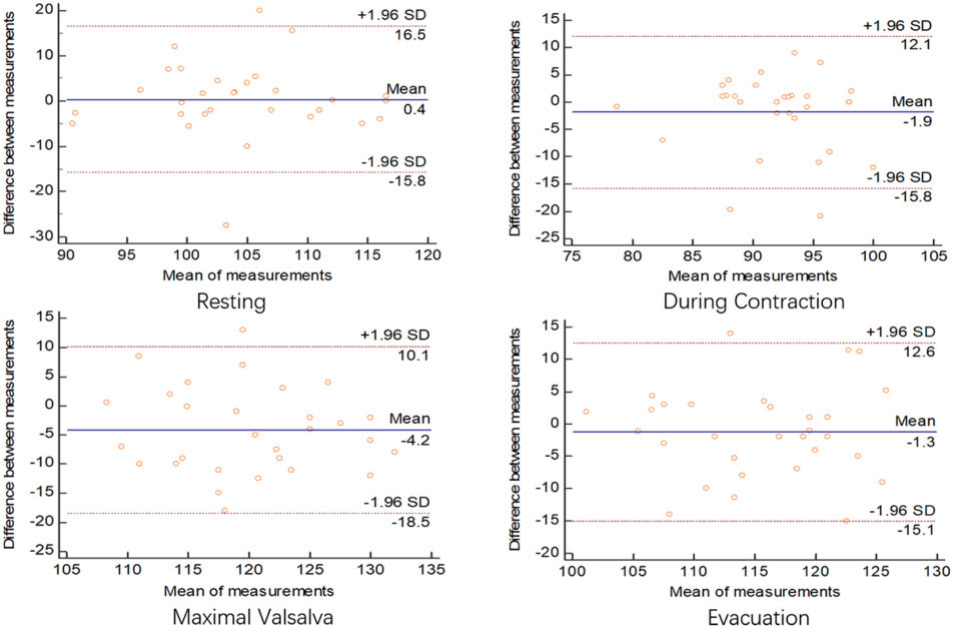
Consistency of anorectal angle (degree) between defecography and ultrasonography in sitting position

**Figure 6.**
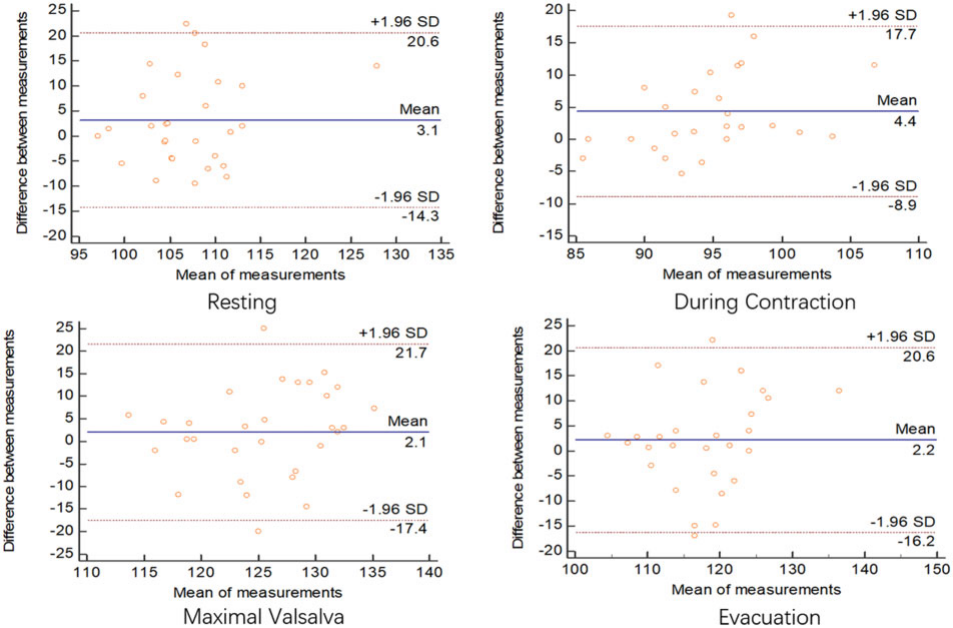
Consistency of anorectal angle (degree) between defecography and ultrasonography in squatting position

**Figure 7.**
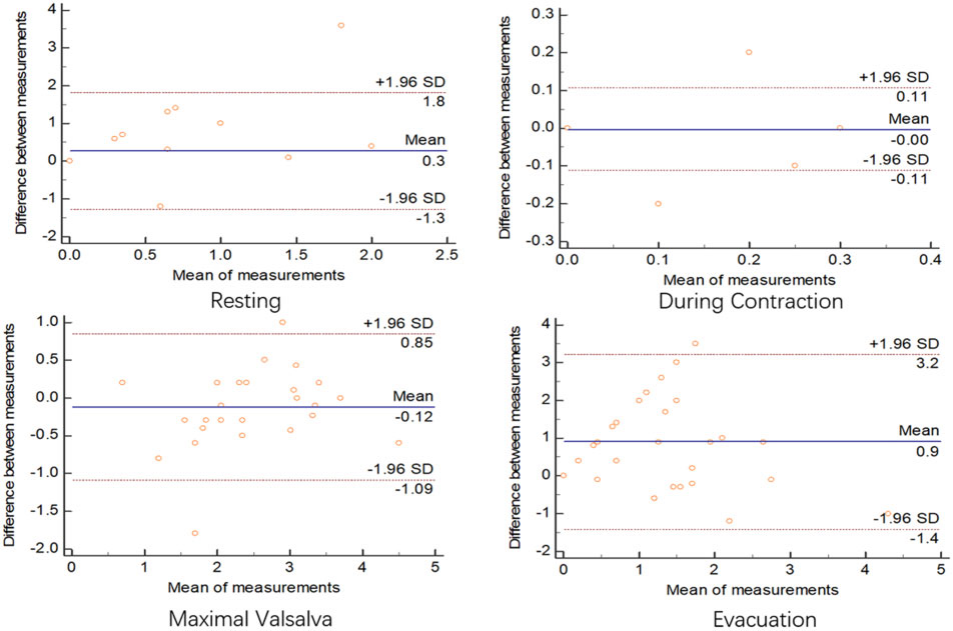
Consistency of depth of rectocele (cm) between defecography and ultrasonography in sitting position

**Figure 8.**
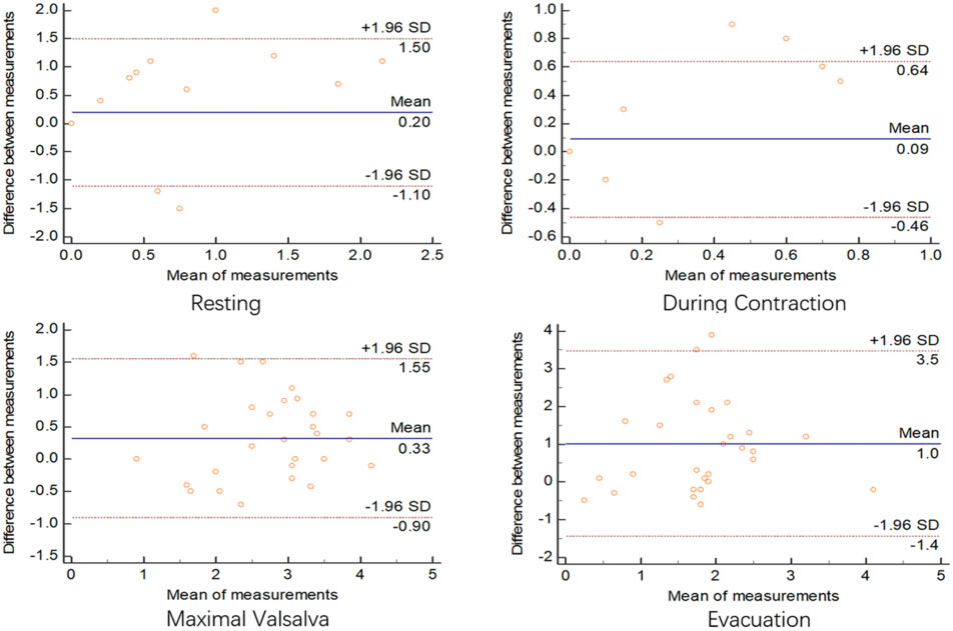
Consistency of depth of rectocele (cm) between defecography and ultrasonography in squatting position

Results from the Bland-Altman semi-quantitative plot showed agreement in the measurement of the anorectal angle and depth of the rectocele between the defecography and ultrasonography methods in both sitting and squatting positions. In the sitting position, the mean differences in the measurement of the anorectal angle between the defecography and ultrasonography methods at four situations varied from –4.2 to 0.4 (Figure 5). Similar differences in the measurement of the anorectal angle between the two methods in the squatting position were also observed, with a mean difference in four situations that varied from 2.1 to 4.4 (Figure 6). The observed differences in the measurement of the depth of the rectocele between the two methods in four situations were smaller than those of the anorectal angle, with mean differences that varied from –0.12 to 0.9 in the sitting position (Figure 7) and from 0.09 to 1.0 in the squatting position (Figure 8). The mean time performed for transperineal ultrasonography was significantly longer than that for defecation proctography (55.43 ± 26.38 vs 40.69 ± 18.25 min, *P *<* *0.05).

## Discussion

This study evaluated compared the consistency of the measurements for rectocele diagnosis via defecography and ultrasonography in varied defecate positions. Our study found the anorectal angle and depth of rectocele between the defecation proctography and transperineal ultrasonography techniques were consistent in both sitting and squatting positions. Although this finding is not in line with previous studies reporting a significant difference in the anorectal angle and depth of rectocele between these two methods, previous studies only examined the defecography method in the sitting position and the ultrasonography method in the left lateral position [9, 19, 20]. Therefore, the position adopted for a patient may play an important role when determining the diagnosis of rectocele via different image methods in clinical practice.

Defecography is the conventional image approach for the diagnosis of rectocele [21], although patients have to receive a large dose of ionizing radiation with this approach and it is not safe for fertile women. As such, healthcare practitioners cautiously apply defecography to a patient for rectocele and are seeking a safe and convenient method to replace the defecography method [22]. By contrast, transperineal ultrasonography is a non-invasive approach with a lower cost. This imaging approach could be used for measuring the area of the levator hiatus and the volume of the rectocele, which cannot be obtained via defecography. Another advantage of transperineal ultrasonography is the ability to strain with a sitting or squatting position, although there is some reticence in doing so in a left lateral position, although the time to perform the transperineal ultrasonography was longer than that for the defecation proctography. It evaluated three positions and measured the area of the levator hiatus and the volume of the rectocele. This technique was complex and involved learning curves. In addition, participants in our study indicated that, compared to the squatting position, the sitting position was more convenient and comfortable when performing both defecography and ultrasonography, although the assessment of other anomalies (e.g. minor perineocele, disruption, or distal rectovaginal septum) with the technology was limited.

Three limitations should be considered in interpreting the findings of this study. First, a small number of participants were included from the outpatient clinic of one hospital, which may make the statistical-analysis results relatively speculative. Second, the level of the perineum descending was not examined by ultrasonography in this study due to the fact that the perineum descending can only be obtained when making the ultrasound probe contact the perineum and keeping pressure on the perineum. Finally, data were only collected from the volunteers with a rectocele, which did not compare with normal female volunteers without any pelvic-floor pathology. However, this is the pilot study exploring the role of the normal defecate position for rectocele diagnosis and the consistency of anorectal angle and depth of rectocele values between the defecography and ultrasonography methods for the rectocele. An observer-blinded high-quality clinical trial will be required to investigate the use of transperineal ultrasonography in the wider pelvic-floor setting in future research. The finding of this study may be used for the basis of future studies exploring the optimal rectocele image diagnostic method.

In conclusion, our results provide preliminary evidence to support the use of transperineal ultrasonography in sitting and squatting positions as a safer imaging test option for rectocele diagnosis. Transperineal ultrasonography is an alternative that correlates satisfactorily with the findings obtained by defecography, provides important data in the evaluation of rectoceles, and offers safety and comfort to patients. Further research with a larger sample size is needed to explore the physiological examination position in the future.

## Authors’ Contributions

Study concept, study design, and drafting of the manuscript: C.W. Material preparation, data collection, and analysis: Y.B.Y., H.Q.Y., H.J.W., and H.T.L. Statistical analysis: B.W. All authors read and approved the final manuscript.

## Funding

This study was funded by the National Natural Science Foundation of China [81603618, 81603625] and Shanghai Municipal Health Commission [2018BR19].

## References

[1] Wexner SD , Zbar AP, Pescatori M, (eds). Complex Anorectal Disorders: Investigation and Management. New York: Springer, 2005, p. 446.

[2] Beck DE , Wexner SD, Rafferty JF, (eds). Gordon and Nivatvongs’ Principles and Practice of Surgery for the Colon, Rectum, and Anus, 4th edn. New York: Thieme, 2019, p. 834.

[3] Fazio VW , Church JM, Delaney CP, Kiran CP (eds). Current Therapy in Colon and Rectal Surgery Third Edition. Philadelphia: Elsevier, 2017, p. 114.

[4] Deng Q , Yu KL, Liu ZY et al Outcomes of a modified Bresler procedure for the treatment of rectocele with rectal intussusception. Gastroenterol Rep (Oxf) 2020;8:457–64. 33442479 10.1093/gastro/goaa027 PMC7793192

[5] Saclarides TJ , Brubaker LT, Altringer WE et al Clarifying the technique of four-contrast defecography. Dis Colon Rectum 1996;39:826. 10.1007/BF02054452 8674379

[6] Martin-Martin GP , Garcia-Armengol J, Roig-Vila JV et al Magnetic resonance defecography versus videodefecography in the study of obstructed defecation syndrome: is video defecography still the test of choice after 50 years? Tech Coloproctol 2017;21:795–802. 28755255 10.1007/s10151-017-1666-0

[7] Beer-Gabel M , Teshler M, Barzilai N et al Dynamic transperineal ultrasound in the diagnosis of pelvic floor disorders. Dis Colon Rectum 2002;45:239–48. 11852339 10.1007/s10350-004-6155-7

[8] Kleinübing H , Pinho MSL, Pescatori M, Regadas FSP, Zbar AP (eds). Transperineal Ultrasonography of Pelvic Floor and Anorectal Anatomy: Technique and Images. Milan: Springer, 2008.

[9] Perniola G , Shek C, Chong CCW et al Defecatioin proctography and translabial ultrasound in the investigation of defecatory disorder. Ultrasound Obstet Gynecol 2008;31:567–71. 18409183 10.1002/uog.5337

[10] Dietz HP , Steensma AB. Posterior compartment prolapse on two-dimensional and tree-dimensional pelvic floor ultrasound: the distinction between true rectocele, perineal hypermobility and enterocele. Ultrasound Obstet Gynecol 2005;26:73–7. 15973648 10.1002/uog.1930

[11] Broekhuis SR , Kluivers KB, Hendriks JCM et al POP-Q, dynamic MR imaging, and perineal ultrasonography: do they agree in the quantification of female pelvic organ prolapse? Int Urogynecol J 2009;20:541–9. 10.1007/s00192-009-0821-1 19221680

[12] Takano S , Sands DR. Influence of body posture on defecation: a prospective study of “The Thinker” position. Tech Coloproctol 2016;20:117–21. 26690926 10.1007/s10151-015-1402-6

[13] Rodriguez-Mias NL , Subramaniam N, Friedman T et al Prolapse assessment supine and standing: do we need different cutoffs for “significant prolapse”? Int Urogynecol J 2018;29:685–9. 28444408 10.1007/s00192-017-3342-3

[14] Chevillotte T , Coudert P, Cawley D et al Influence of posture on relationships between pelvic parameters and lumbar lordosis: comparison of the standing, seated, and supine positions: a preliminary study. Orthop Traumatol Surg Res 2018;104:565–8. 30009961 10.1016/j.otsr.2018.06.005

[15] Sultan AH , Monga A, Lee J et al An International Urogynecological Association (IUGA)/International Continence Society (ICS) joint report on the terminology for female anorectal dysfunction. Int Urogynecol J 2017;28:5–31. 27774569 10.1007/s00192-016-3140-3

[16] Altomare DF , Spazzafumo L, Rinaldi M et al Set-up and statistical validation of a new scoring system for obstructed defaecation syndrome. Colorectal Dis 2008;10:84–8. 17441968 10.1111/j.1463-1318.2007.01262.x

[17] Agachan F , Chen T, Pfeifer J et al Constipation scoring system to simplify evaluation and management of constipated patients. Dis Colon Rectum 1996;39:681–5. 8646957 10.1007/BF02056950

[18] Lewis SJ , Heaton KW. Stool form scale as a useful guide to intestinal transit time. Scan J Gastroenterol 1997;32:920–4. 10.3109/00365529709011203 9299672

[19] Beer-Gabel M , Teshler M, Schechtman E et al Dynamic transperineal ultrasound vs. defecography in patients with evacuatory difficulty: a pilot study. Int J Colorectal Dis 2004;19:60–7. 12761642 10.1007/s00384-003-0508-x

[20] Shorvon PJ , McHugh S, Diamant NE et al Defecography in normal volunteers: results and implications. Gut 1989;30:1737–49. 2612988 10.1136/gut.30.12.1737 PMC1434461

[21] Bharucha AE , Dorn SD, Lembo A, American Gastroenterological Association et al American Gastroenterological Association Medical Position Statement on Constipation. Gastroenterology 2013;144:211–7. 23261064 10.1053/j.gastro.2012.10.029

[22] Felt-Bersma RJ , Luth WJ, Janssen JJ et al Defecography in patients with anorectal disorders: which findings are clinically relevant? Dis Colon Rectum 1990;33:277–84. 2323276 10.1007/BF02055468

